# Interaction Between Konjac Glucomannan and Gut Microbiota and Its Impact on Health

**DOI:** 10.3390/biology14080923

**Published:** 2025-07-23

**Authors:** Yufen Yu, Shuo Jin, Yi Yang, Xiaodong Han, Rongfa Guan, Hao Zhong

**Affiliations:** 1 College of Food Science and Technology, Zhejiang University of Technology, Hangzhou 310014, China; 2 Rawbery Foods Biotech Co., Ltd., Huzhou 313099, China; 3 Sichuan Sweet Agriculture Co., Ltd., Guangyuan 628475, China

**Keywords:** konjac glucomannan, gut microbiota, short-chain fatty acids, disease

## Abstract

Konjac glucomannan (KGM) is a natural polysaccharide polymer. It is degraded by gut microbiota-derived β-mannanase into small-molecule nutrients, which exert diverse physiological regulatory effects. As a prebiotic, KGM modulates gut microbiota composition. It selectively fosters the proliferation of beneficial commensals and suppresses potential pathogens, thereby alleviating microbiota-related disorders. Moreover, microbiota fermentation of KGM produces metabolites. Short-chain fatty acids (SCFAs) are particularly notable among these metabolites. They exert multifaceted beneficial effects, including metabolic regulation, intestinal barrier strengthening, and neuroprotective functions. These effects are mediated through inhibition of inflammatory pathways (e.g., NF-κB, MAPK), modulation of lipid metabolism genes (e.g., CD36), and regulation of neurotransmitters (e.g., GABA, 5-HT). This highlights KGM’s therapeutic potential for metabolic, inflammatory, and neurodegenerative diseases. Current clinical use is limited by dose-dependent adverse effects and interindividual response variability, which stem from different microbial communities. This necessitates personalized dosage strategies. Despite these limitations, KGM as a prebiotic polysaccharide exhibits multifaceted bioactivity. Current evidence suggests its potential to synergistically modulate metabolic pathways, gut microbiota composition, immune cell signaling, and neuroendocrine interactions. This highlights its promise for developing novel therapeutic interventions.

## Simple Summary

Konjac glucomannan (KGM), a highly effective prebiotic, exerts a pivotal influence on regulating the composition and structure of gut microbiota. An increasing body of evidence underscores the robust correlation between gut microbial diversity and human health, encompassing its significance in a wide array of diseases. This review delves into the structural features of KGM and its impact on gut microbiota. Particular emphasis is placed on the mechanisms through which KGM-mediated microbial modulation mitigates metabolic disorders, intestinal diseases, and neurological disorders, thus exploring its potential as a novel therapeutic approach.

## 1. Introduction

For modern high-risk diseases—including metabolic disorders, inflammatory bowel diseases, autoimmune conditions, and neurodegenerative disorders—current treatment strategies (e.g., insulin sensitizers and anti-inflammatory drugs) have shown some efficacy, but long-term effectiveness and safety remain major challenges [1]. Despite their heterogeneous clinical manifestations, growing evidence suggests that their pathogenesis is closely linked to gut microbiota dysbiosis. The gut microbiome is a “superorganism” composed of trillions of microorganisms [2]. It show a highly dynamic and diverse structure, mainly composed of Firmicutes, Bacteroidetes, Actinobacteria, Proteobacteria, and Verrucomicrobia [3]. In healthy adults, Firmicutes is the most abundant phylum, followed by Bacteroidetes and Actinobacteria. Under normal conditions, the gut microbiota maintains a symbiotic relationship with the host, regulating essential physiological functions, such as nutrient digestion and absorption, protection against pathogens, and immune homeostasis [4]. However, gut dysbiosis disrupts this symbiotic balance and may lead to various physiological dysfunctions. Extensive research demonstrates that gut microbiota dysbiosis can exacerbate disease progression through multiple mechanisms, including the following: (1) Dysregulation of energy metabolism; (2) compromised intestinal barrier integrity; (3) disruption of immune system homeostasis; and (4) impaired brain neuronal function [5,6,7]. Thus, dietary interventions targeting gut microbiota modulation hold promise as a novel therapeutic approach for mitigating modern high-risk diseases.

*Amorphophallus konjac* is rich in alkaloids, starch, proteins, and soluble polysaccharides [8]. Its tubers contain up to 60% konjac glucomannan (KGM), a unique natural polysaccharide [9]. KGM is extracted by physical or chemical methods (e.g., grinding, water extraction, alcohol precipitation) and consists of D-glucose and D-mannose. It exhibits high viscosity, gel-forming capacity, and prebiotic activity, with benefits including blood glucose/lipid reduction, intestinal barrier enhancement, immune modulation, and low caloric value [10,11]. Recent studies have further revealed that KGM selectively promotes beneficial bacteria (e.g., *Bifidobacteria*, *Lactobacilli*), inhibits harmful strains (e.g., *Escherichia coli*), optimizes the structure of gut microbiota, and exerts multi-system health effects [12]. Moreover, as a non-digestible polysaccharide, KGM is metabolized by gut microbes into absorbable small molecules and SCFAs, thereby improving gut barrier function and systemic metabolism [13,14]. Its water retention capacity also enhances satiety, slows glucose/lipid absorption, and reduce obesity risks [15]. A thorough comprehension of these mechanisms will underpin the development of novel dietary therapeutics for modern high-risk disease management.

Current research has mainly focused on the therapeutic effects of KGM in specific diseases, but there is still a lack of research on the mechanism of its systemic health effect through the regulation of gut microbiota. This review synthesizes the latest evidence to comprehensively elucidate the KGM–gut microbiota interaction, the process of gut microbiota fermentation and metabolism of KGM, and how KGM mediates the mediation mechanism of gut microbiota to alleviate metabolic disorders, intestinal inflammation, and neurological diseases. It provides a new perspective for clinical management of modern high-risk diseases.

## 2. Chemical Composition and Structural Characteristics of KGM

**Figure 1:**
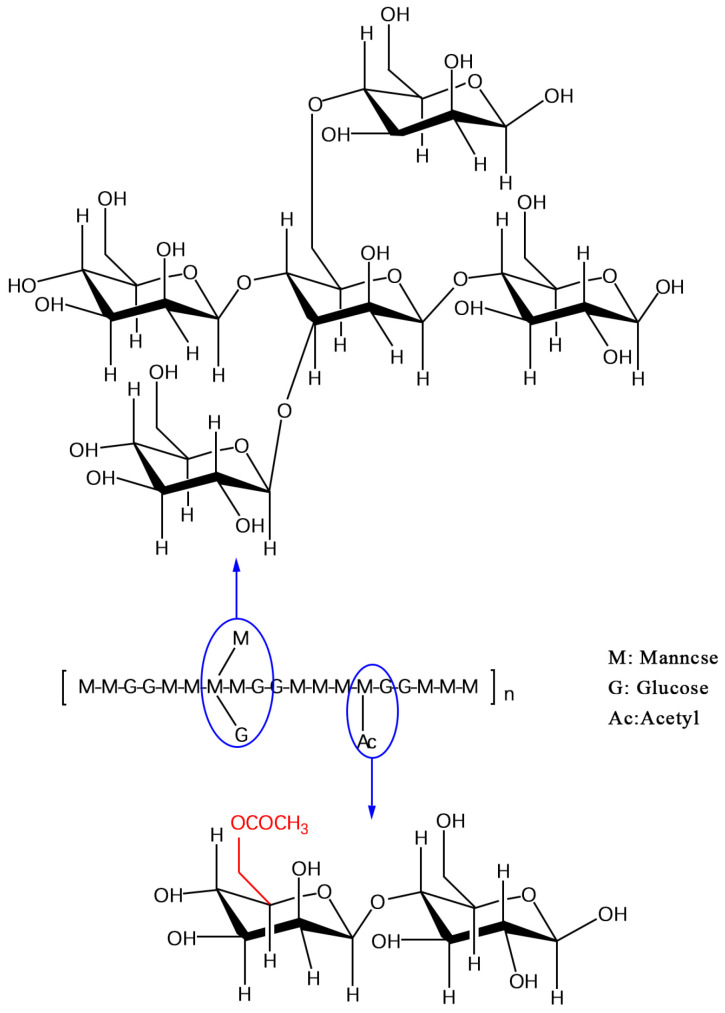
KGM molecular structure formula; M-M-G sequence consists of a backbone formed by glucose (G) and mannose (M) linked via β-1,4 glycosidic bonds, with β-1,6-glucosyl branches. In the Ac-M-G sequence, acetyl groups are randomly attached to the C-6 position of mannose (M) units. Adopted from [10].

KGM is a natural polysaccharide that can be hydrolyzed into D-glucose and D-mannose under specific conditions, with a molecular formula of (C_6_H_10_O_5_)_n_ [16]. The main chain of KGM is composed of two monosaccharides linked by β-1,4 glycosidic bonds, with a molar ratio of 1:1.6-1:2. The branched chain, consisting of mannose, is connected to the C3 position of the main chain through β-1,3 glycosidic bonds [17]. KGM exhibits a broad molecular weight range, typically between 200 kDa and 2000 kDa [18]. The molecular weight distribution of KGM is influenced by various factors, including different Konjac varieties (such as *Amorphophallus albus* and *Amorphophallus konjac*) and extraction methods (including acid, alkali, and enzyme methods) [12,19]. Furthermore, acetyl groups, which are crucial for KGM’s solubility and gelation properties, are attached to the C6 position via ester bonds (Figure 1). On average, one acetyl group is present for every 9–20 sugar residues, resulting in a degree of substitution of approximately 5–10% in the main chain [10,20].

KGM is a high-molecular-weight polysaccharide with a complex structure. Its degradation primarily relies on the synergistic action of β-mannanase, β-mannosidase, and β-glucosidase [21]. The enzymatic process proceeds as follows: (1) β-mannanase cleaves the long-chain KGM polysaccharide into shorter mannan oligosaccharides (MOSs) and glucose oligosaccharides (GOSs) [22]; (2) β-mannosidase hydrolyzes the β-1,4-mannosidic bond at the non-reducing ends of the MOS fragments, releasing mannose [23]; (3) concurrently, β-glucosidase hydrolyzes the β-1,4-glucosidic bond at the non-reducing ends of the GOS fragments, yielding glucose [24]. Notably, humans lack digestive enzymes capable of directly degrading KGM, rendering it indigestible by the host. However, studies have identified KGM-degrading gut microbiota that produce β-mannanase in the small intestine and colon [25]. Specifically, *Bifidobacterium* and *Bacteroides* strains have been confirmed to encode β-mannanase, enabling them to metabolize KGM [26,27]. Through enzymatic degradation, these microbes convert KGM into SCFAs—compounds that serve as an energy source for the host and mediate diverse physiological effects [28].

## 3. Regulatory Effects of KGM on Gut Microbiota

### 3.1. Changes in Gut Microbiota Composition

**Figure 2:**
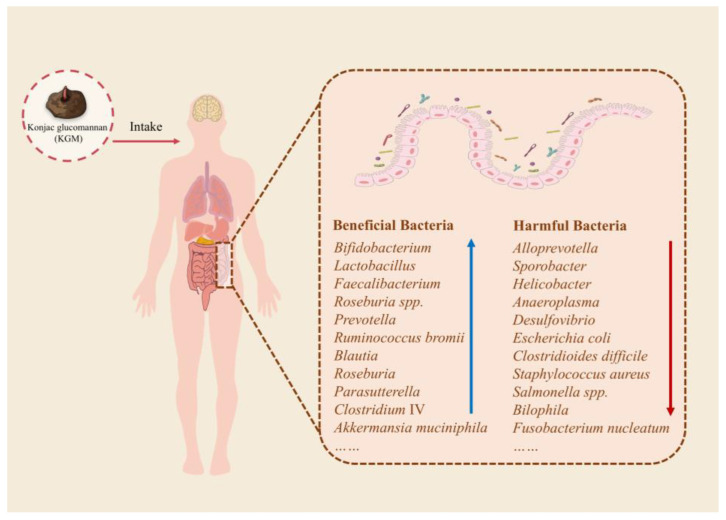
Effect of KGM on the structure of gut microbiota; “↑” indicates increases, while “↓” indicates decreases.

As illustrated in Figure 2, KGM effectively modulates gut microbiota homeostasis by selectively promoting the proliferation of beneficial bacterial species while suppressing pathogenic strains, thereby improving intestinal microecological health.

#### 3.1.1. The Promoting Effect of KGM on Beneficial Bacteria

The gut microbiota plays a crucial role in human physiological processes and is a key determinant of intestinal barrier integrity. Beneficial gut bacteria not only inhibit the proliferation of pathogenic bacteria but also neutralize harmful metabolites that threaten host health. Moreover, their enrichment of beneficial bacteria contributes to maintaining gut microbiota homeostasis, thereby preventing intestinal barrier dysfunction. Notably, KGM supplementation has been shown to promote the growth of beneficial intestinal bacteria and restore microbial balance, potentially alleviating metabolic disorders. Liu et al. demonstrated in an irradiated mice model that KGM effectively mitigated radiation-induced gut microbiota dysbiosis and significantly increased the abundance of probiotics, including *Lactobacillus*, *Lachnoclostridium*, *Alloprevotella*, *Blautia*, and *Akkermansia muciniphila* [29]. Among these, *Lactobacillus* and its metabolites exhibit therapeutic potential against various gastrointestinal diseases [30]. *Akkermansia muciniphila* enhance insulin sensitivity and glucose tolerance through anti-inflammatory mechanism, offering protective effects against type 2 diabetes mellitus (T2DM) [31]. Supporting these findings, Hong et al. revealed that dietary supplementation with 100 g/kg of KGM in high-fat diet (HFD) mice for 16 weeks markedly increased the abundance of *Akkermansia muciniphila*, *Alistipes*, *Olsenella*, and *Bifidobacterium* [32]. As a dominant commensal bacterium in the human gut, *Bifidobacterium* ferments KGM to produce acetate and lactic acid, thereby lowing intestinal pH and suppressing pathogenic bacterial growth [33,34]. Furthermore, multiple studies report that KGM enhances the abundance of *Prevotella*, *Roseburia*, *Faecalibacterium*, and *Ruminococcus* [28,35]. These microbial shifts improve the intestinal microecological equilibrium, strengthen barrier function, and attenuate inflammatory response.

#### 3.1.2. The Inhibitory Effect of KGM on Potentially Harmful Bacteria

KGM and its metabolites directly modulate the internal microenvironment, altering the structure and composition of the gut microbiota. Notably, KGM restores microbial equilibrium primarily through a prebiotic effect, indirectly suppressing pathogenic bacteria rather than exerting direct bactericidal activity. For instance, propionate, a degradation product of KGM, exhibits potent growth-inhibiting effects against *Salmonella enterica*. Additionally, organic acids derived from KGM metabolism lower intestinal pH, thereby creating an unfavorable environment for pathogenic bacterial proliferation. Gao et al. demonstrated that maternal KGM supplementation during late pregnancy and lactation significantly reduced the abundance of *unclassified Erysipelotrichaceae*, *Clostridium*, and *Candidatus Saccharimonas* [36]. Of particular clinical relevance, *Candidatus Saccharimonas* is associated with inflammatory conditions and dysregulated immune responses, and its enrichment has been observed in patients with Crohn’s disease (CD) and ulcerative colitis (UC) [37]. Further evidence indicates that KGM and depolymerized KGM (DKGM) treatment in HFD mice markedly decreased the relative abundance of opportunistic pathogens, including *Sporobacter*, *Helicobacter*, *Anaeroplasma*, and *Desulfovibrio* [32]. The excessive proliferation of *Desulfovibrio* is particularly detrimental, as it releases endotoxins (e.g., lipopolysaccharides, LPS), compromises intestinal barrier integrity, disrupts metabolic balance, and perpetuates chronic inflammation, ultimately contributing to systemic pathophysiology [38]. Beyond its metabolic effects, KGM’s gel-forming properties physically encapsulate pathogenic bacteria, reducing their adhesion to the intestinal epithelium and mitigating subsequent barrier damage.

### 3.2. Metabolites of KGM Fermented by Gut Microbiota

**Figure 3:**
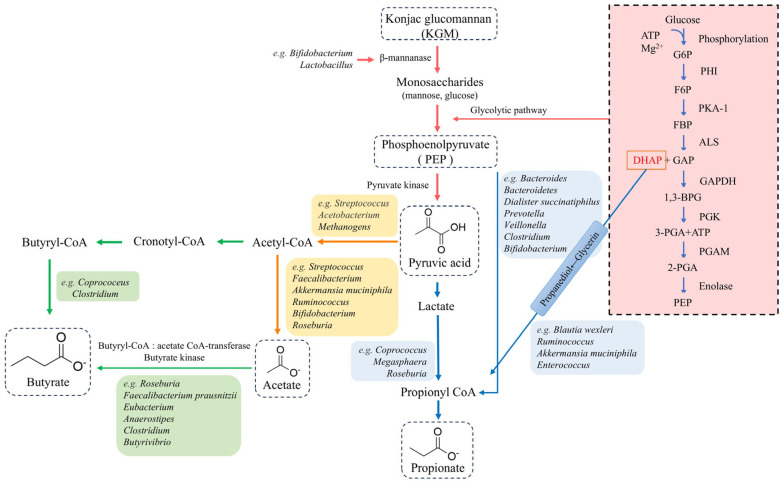
The pathway of KGM metabolized into SCFAs by gut microbiota in vivo; the representative gut microbiota involved in each metabolic step are marked in the diagram, and the bacterial division and metabolic flow trend of KGM to acetate, propionate, and butyrate were analyzed.

Currently, SCFAs represent the most extensively studied metabolites in gut microbiota research. These compounds are predominantly produced through microbial fermentation of indigestible polysaccharides—including resistant starch, oligofructose, and various monosaccharides and polysaccharides—by gut microbiota in the cecum and colon [39,40]. These SCFAs, primarily comprising acetate, propionate, valerate, and butyrate, serve as crucial energy sources when metabolized in the mitochondrial matrix, providing fuel for diverse tissues, such as liver, heart, and skeletal muscle [41]. Beyond their metabolic roles, SCFAs exhibit multifaceted physiological functions. These include blood glucose regulation, controlled drug release through hybridization with monosaccharide molecules, maintenance of water and electrolyte balance, antimicrobial and anti-inflammatory activities, modulation of gut microbiota composition, enhancement of intestinal function, immune regulation, antitumor effects, and gene expression modulation [42]. Importantly, these diverse functions highlight the systemic impact of SCFAs on host physiology. KGM, a natural polysaccharide derived from the konjac plant [43], represents a particularly interesting substrate for SCFAs production. Due to the absence of specific digestive enzymes in humans, KGM resists direct digestion in the gastrointestinal tract and requires fermentation by colonic bacteria, such as *Lactobacillus* and *Bifidobacterium*, to exert its health benefits [13,32]. This fermentation process primarily yields acetate, propionate, and butyrate [44], making KGM an important dietary component for SCFA production. As shown in Figure 3, colonic bacteria expressing β-mannanase initially hydrolyze KGM into monosaccharides (pentose and hexose), which are subsequently converted to pyruvate via glycolytic pathways including the Embden–Meyerhof–Parnas (EMP), pentose phosphate (HMP), and Entner–Doudoroff (ED) pathway [33,45]. Supporting this metabolic cascade, Qi et al.’s metabolomics study demonstrated significantly elevated pyruvate levels in the gastrointestinal tract of constipated mice following KGM supplementation [46]. As a key intermediate product in the metabolic pathway of KGM fermentation, pyruvate synthesizes SCFAs through the following three pathways: (1) acetate production via acetyl-CoA or the Wood–Ljungdahl pathway; (2) propionate generation through succinic acid, acrylate, or propanediol pathways; and (3) butyrate synthesis via butyrate kinase, butyryl-CoA–acetate CoA transferase, or acetyl-CoA acetyltransferase pathways [47].

Notably, KGM fermentation by gut microbiota generates not only SCFAs but also gases and organic acids. These byproducts collectively contribute to intestinal environmental optimization, demonstrating that KGM’s impact extends beyond SCFA production to exert profound effects on gut health and systemic physiological functions [48,49].

#### 3.2.1. Acetate

Acetate plays a crucial role in maintaining intestinal microecological homeostasis and promoting systemic health [50]. As the most abundant SCFAs in the colon, acetate serves dual functions as both a metabolic substrate and signaling molecule. It provides an essential energy source for beneficial gut microbiota, supporting their proliferation and thereby contributing to the stability of the intestinal microbial ecosystem [51]. The antimicrobial properties of acetate constitute another important mechanism of action. By lowering intestinal pH, acetate creates an unfavorable environment for pathogenic bacteria, such as *Clostridium* spp., significantly reducing their colonization potential and infection risk [52,53]. Beyond its microbial modulatory effects, acetate enhances intestinal epithelial integrity through multiple mechanisms [54]. Pioneering work by Macia et al. using *Gpr43* −/− and *GPR109a* −/− mice models demonstrated that acetate directly activates nucleotide-binding oligomerization domain-like receptor protein 3 (NLRP3) inflammasome, stimulating IL-18 secretion. The released IL-18 subsequently binds to its cognate receptor on intestinal epithelial cells, reinforcing intestinal barrier function—a critical mechanism in colitis prevention [55]. Notably, acetate’s physiological influence extends beyond the gastrointestinal tract to systemic metabolic regulation. Kimura et al. revealed that acetate mediates metabolic regulation through GPR43 signaling in adipocytes. By inhibiting the insulin signaling pathway, acetate reduces lipid accumulation in adipose tissue while promoting the utilization of free fatty acids and glucose in peripheral tissues [56]. This dual action positions acetate as a potential therapeutic target for metabolic disorders.

#### 3.2.2. Propionate

Propionate, a three-carbon SCFA, is primarily synthesized by intestinal microbiota belonging to the Bacteroidetes and Firmicutes phyla, particularly members of the Lachnospiraceae family, through both the succinic acid and propylene glycol pathways [57]. This metabolite plays a pivotal role in maintaining intestinal homeostasis by enhancing epithelial integrity and barrier function, thereby exerting protective effects against inflammatory bowel diseases, such as CD and colorectal cancer (CRC) [58]. At the molecular level, propionate modulates immune responses through dual mechanisms, namely (1) inhibition of histone deacetylases (HDACs), which regulates inflammation-associated gene expression; (2) activation of specific G protein-coupled receptors (GPCRs) that influence cellular metabolism [42]. Beyond its local intestinal effects, propionate acts as an important signaling molecule that contributes to systemic metabolic regulation by promoting hepatic gluconeogenesis and maintaining lipid–glucose homeostasis through adenosine monophosphate-activated protein kinase-dependent pathways [59]. Additional research by Edward S et al. found that propionate enhances glucose-stimulated insulin secretion via protein kinase C (PKC) signaling, improving β-cell function and ameliorating metabolic disorders [60]. In addition, propionate influences energy homeostasis through the gut–brain axis by stimulating the secretion of anorexigenic hormones, including peptide YY (PYY) and glucagon-like peptide-1 (GLP-1), thereby regulating satiety signals [61].

#### 3.2.3. Butyrate

In general, polysaccharides produce butyrate through bacterial fermentation in the host colon through two metabolic pathways, namely (1) phosphorylation of butyryl-CoA to form butyryl phosphate, which is converted to butyrate by butyrate kinase [62], and (2) transfer of the CoA group from butyryl-CoA to acetate via butyryl-CoA–acetate-CoA transferase, yielding butyrate and acetyl-CoA [63]. As the preferred energy source for colonic epithelial cells, butyrate critically supports intestinal homeostasis [64]. Among SCFAs, butyrate stands out due to its diverse physiological functions, including the facilitation of transepithelial transport, attenuation of mucosal inflammation, reduction of oxidative stress, enhancement of epithelial barrier integrity, and prevention of CRC [65]. The predominant butyrate-producing bacteria, primarily *Clostridium*-related genera within the Firmicutes phylum (e.g., *Aecalibacterium*, *Roseburia*, *Eubacterium*, *Anaerostipes*, *Coprococcus*, *Subdoligranulum*, and *Anaerobutyricum*), underscore its microbial origins [66]. Beyond gut health, butyrate demonstrates promise in metabolic diseases. For instance, Gao et al. observed significantly reduction in body weight, adiposity, fasting glucose, and insulin resistance in obese mice after 5 weeks of supplementation, highlighting its potential against diet-induced metabolic disorders [67]. Moreover, butyrate exerts potent anti-inflammatory effects by suppressing pro-inflammatory cytokines (IFN-γ, TNF-α, IL-1B, IL-6 and IL-8) while elevating anti-inflammatory mediators (IL-10 and TGF-B), thereby modulating immune responses [68,69]. Additionally, it fortifies intestinal defense by stimulating goblet cell mucin production and tightening epithelial junctions, restricting systemic translocation of luminal pathogens and toxins [70].

## 4. The Health Effects of KGM Mediated by Gut Microbiota

**Figure 4:**
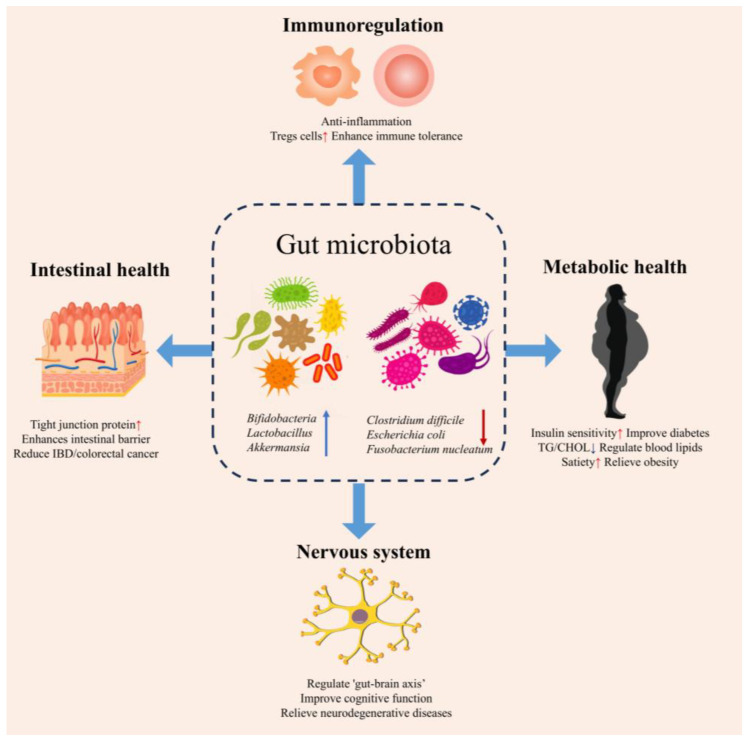
Beneficial effects of intestinal flora in health; “↑” indicates increases, while “↓” indicates decreases.

As shown in Figure 4, the gut microbiota coordinates many physiological processes, and the dysbiosis of this microbial community is related to the pathogenesis of various disorders. Notably, KGM has demonstrated significant regulatory effects on gut microbiota composition and function. Through microbial fermentation, KGM promotes the production of bioactive metabolites, particularly SCFAs, which mediate its beneficial physiological effects. As summarized in Table 1, KGM modulates gut microbiota–host interactions through multiple mechanisms, offering therapeutic potential for various human diseases.

### 4.1. Metabolic Disorders

#### 4.1.1. Obesity

Obesity is a complex metabolic disorder that extends beyond weight gain, significantly increasing the risk of diabetes, cardiovascular disease, and immune-related diseases [79]. As a natural functional polysaccharide, KGM demonstrates pronounced anti-obesity properties through its remarkable water-binding capacity and high viscosity, mechanisms which collectively promote satiety induction via gastric distension, delay gastric emptying, attenuate postprandial glycemic fluctuations, and improve dyslipidemia profiles [80,81,82].

Importantly, KGM’s recently elucidated modulatory effect on gut microbiota composition further expand its therapeutic potential, positioning it as a multifaceted intervention agent in comprehensive obesity management strategies. In general, there is a close relationship between obesity and gut microbiota dysbiosis. The diversity and richness of gut microbiota in obese individuals tend to decrease [83], and the ratio of Firmicutes to Bacteroidetes (F/B) is significantly increased [84]. This dysbiosis is considered to be an important driver of obesity and its related metabolic disorders. Guo et al. found that KGM supplementation not only reduced the F/B ratio in HFD-induced obese mice but also significantly increased the abundance of Rikenellaceae, Bacteroidaceae, and Akkermansiaceae in the intestine tract [85]. These microbial taxa are well known to be closely associated with metabolic health and immune regulation. Specifically, Akkermansiaceae abundance is negatively correlated with the incidence of obesity-related metabolic disorders [86], while Rikenellaceae abundance is positively correlated with appetite suppression, increased energy expenditure, and inhibition adipogenesis [87]. Thus, regulating the abundance of this gut microbiota may directly ameliorate obesity. Further evidence highlights the breadth of KGM’s microbial modulation, Liu et al. induced obesity in mice with a high-fat and high-fructose diet (HFFD) and conducted a 12-week KGM intervention. They observed that KGM intake significantly altered the β diversity of gut microbiota in obese mice, specifically enhancing the abundance of *Clostridium* IV and *Parasutterella* (linked to cholesterol metabolism) while suppressing *Coprobacter* and *Streptococcus* (associated with inflammation) [88]. Structural optimization likely improves obesity by modulating SCFAs metabolism and reducing intestinal inflammation. Notably, *Parasutterella* not only participates in bile acid homeostasis and cholesterol metabolism but also modulates hypothalamic inflammation in obesity, thereby influencing appetite and satiety regulation and effectively improving obesity [89].

Furthermore, KGM intake significantly reduced body weight, fat mass, and blood lipid levels in obese mice while alleviating obesity-induced liver damage and inflammation. Collectively, these findings demonstrate that KGM effectively restores gut microbiota composition, mitigates dysbiosis in obesity, and subsequently regulates lipid metabolism, highlighting its multifaceted therapeutic potential.

#### 4.1.2. Diabetes

Diabetes is a chronic metabolic disorder characterized by hyperglycemia [90]. Accumulating evidence in recent years suggests that gut microbiota plays a pivotal role in the pathogenesis and progression of diabetes, with alterations in its composition and function potentially contributing directly or indirectly to the diabetes [91]. In healthy individuals, the gut microbiota maintains high diversity and dynamic equilibrium, whereas in diabetic patients, this balance is often broken [92]. Specifically, the abundance of beneficial bacteria, such as *Bifidobacterium* and *Faecalibacterium prausnitzii*, is markedly reduced in T2DM patients compared to healthy individuals [93,94], whereas the abundance of opportunistic pathogens, like *Bacteroides*, *Escherichia coli*, and *Desulfovibrio*, is significantly increased [95]. *Bifidobacterium* and *Faecalibacterium prausnitzii* ferment dietary fibers to produce SCFAs. These SCFAs can not only enhance insulin sensitivity and glucose metabolism but also exert potent anti-inflammatory effects, thereby contributing to diabetes prevention and management [96,97].

KGM, as a natural functional dietary fiber, exhibits anti-diabetic properties through its modulatory effects on gut microbiota and subsequent production of beneficial metabolites. Evidence indicates that viscous dietary fiber consumption effectively elevates the relative abundance of probiotics, including *Enterococcus* and lactic acid bacteria [98]. In an experimental study by Deng et al., dietary supplementation with KGM in HFD-fed mice demonstrated significant enhancement of gut microbiota diversity, with particular enrichment of *Ruminococcus* and *Clostridium* genera. This microbial remodeling was concomitant with increased SCFA production and upregulated G protein-coupled receptor (GPCR) expression [73]. These changes further improved the regulation of bile acid synthesis, thereby contributing to the anti-diabetic effects of KGM. Clinical trials validate KGM’s translational potential: In a 28-day intervention study by Chen et al., 22 diabetic subjects consumed 3.6 g of KGM daily. This supplementation significantly increased fecal excretion of neutral sterols and bile acids while mitigating postprandial blood glucose elevation, demonstrating KGM’s therapeutic potential in diabetes [99].Beyond bile acids, KGM targets amino acid metabolism, mechanistic studies reveal that KGM’s therapeutic potential may involve suppression of microbial taxa associated with branched-chain amino acids (BCAA), including *Clostridium*, *Bacteroides*, *Prevotella*, *Klebsiella*, and *Streptococcus* [100]. This is clinically significant, because elevated BCAA levels are known to activate the mTOR-S6K1 signaling axis in hepatic, muscular, and adipose tissues, where sustained mTOR activation induces inhibitory serine phosphorylation of IRS-1, ultimately leading to insulin resistance [101]. Recent investigations have additionally demonstrated that coadministration of *Polygonatum cyrtonema* Hua polysaccharide (PCP) with KGM effectively attenuates postprandial glycemic excursions in dysphagic patients receiving liquid nutritional support, primarily through a significant reduction in *S24-7* and *Helicobacter pylori* [74]. These microbial markers are strongly linked to glucose dysregulation, as *S24-7* abundance shows a strong positive correlation with fasting blood glucose (FBG) variability, while *Helicobacter pylori* exhibit significant associations with FBG, insulin levels, and triglyceride (TG) levels. Complementary studies further associate decreased *S24-7* abundance with improved glucose tolerance and document positive correlations between *Helicobacter pylori* and glycated hemoglobin levels [102,103]. Finally, KGM’s promotion of beneficial bacteria offers systemic benefits, as its ability to proliferate *Akkermansia muciniphila* in HFD murine models enhances insulin sensitivity and reduces systemic inflammation [72,104].

Collectively, these findings position KGM as a promising therapeutic agent for diabetes management through its multifaceted mechanisms of action, namely gut microbiota composition modulation, SCFA production enhancement, suppression of BCAA-producing bacteria, and promotion of beneficial microbial taxa. These properties underscore the significant potential of KGM in developing novel strategies for the prevention and treatment of diabetes.

#### 4.1.3. Hyperlipidemia

Hyperlipidemia is defined as the presence of excessive fat or lipids in the blood [105]. Strictly speaking, hyperlipidemia is not an independent disease but a disorder of lipid metabolism [106]. As a water-soluble polysaccharide, KGM has excellent hypolipidemic effect. On the one hand, KGM can encapsulate lipids in gel-like substances through adsorption in the gastrointestinal tract, and these encapsulated lipids are then excreted with feces, thereby reducing lipid absorption [107]. On the other hand, KGM is fermented by gut microbiota in the colon, which not only alters the composition of gut microbiota but also promotes the production of SCFAs (such as acetic acid, propionic acid, and butyric acid) [108]. These SCFAs act on the liver, and other organs through blood circulation, regulating the expression of genes related to lipid metabolism.

Beyond these direct physicochemical effects, KGM’s therapeutic potential is further amplified through systematic modulation of gut microbiota–host metabolic crosstalk. A compelling example comes from studies on metabolic regulation: Supplementation of KGM in HFD-fed mice demonstrated significant improvements in blood lipid and intestinal lipid accumulation. The effect is attributed to KGM‘s ability to modulate gut microbiota composition—specifically, increasing the abundance of *Akkermansia muciniphila* and *Alistipes* and decreasing the abundance of *Allobaculum*, *Acetatifactor*, and *Helicobacter pylori*—accompanied by enhanced SCFA metabolism. The downstream consequences of these changes are profound; SCFAs activate GPR41 and GPR43, inhibiting lipid deposition in adipose tissue. At the molecular level, KGM regulates the Gut-TLR/MyD88-REV-ERBα-NFIL3 signaling axis, downregulates lipid transport genes (CD36/FABP4), and suppresses HDAC3 expression, thereby reducing intestinal lipid absorption and mitigating HFD-induced metabolic dysfunction [32]. Notably, *Helicobacter pylori* abundance is positively correlated with elevated plasma total cholesterol (TC) and TG levels [109], suggesting its detrimental role in lipid metabolism. The reduction in these bacteria by KGM intervention further supports its efficacy in alleviating dyslipidemia. Recent research has identified synergistic combinations that enhance KGM’s effects. The rice starch–KGM (ERS-KGM) combination exhibits a pronounced effect on gut microbiota modulation. In HFD mice, ERS-KGM treatment significantly enhanced microbial diversity and richness, with increased *Olsenella*, *Christensenellaceae*, *Christensenellales*, *Rombuterella*, Enterococcaceae and Eggerthellaceae [110]. These microbial changes translate into functional metabolic benefits through multiple pathways. For instance, Enterococcaceae strains, such as *Enterococcus faecalis*, secrete bile salt hydrolases, which promote bile acid deconjugation and excretion, thereby reducing cholesterol reabsorption [111]. Similarly, Eggerthellaceae contributes to SCFA-mediated regulation by inhibiting lipogenic gene expression [112].

Collectively, KGM ameliorates host lipid metabolism and reduces blood lipid levels by reshaping the gut microbiota composition, offering a promising therapeutic strategy for hyperlipidemia and related metabolic disorders.

### 4.2. Gastrointestinal Disease

#### 4.2.1. Inflammatory Bowel Disease

IBD is a group of chronic and relapsing inflammatory disorders of the gastrointestinal tract, primarily comprising CD and UC [113]. The etiology of IBD is multifactorial, involving genetic susceptibility, environmental triggers, immune dysregulation, and gut microbiota disorders [114,115]. At present, accumulating evidence underscores the pivotal role of gut microbiota composition and diversity in IBD pathogenesis [116,117]. Halfvarson et al. demonstrated that the gut microbiota undergoes significant compositional shifts during early IBD progression, with markedly greater microbial fluctuation in IBD patients compared to healthy individuals [118]. A balanced gut microbiota confers intestinal protection by preserving epithelial barrier integrity, modulating immune responses, and maintaining metabolic homeostasis [119]. Conversely, microbiota dysbiosis in IBD patients disrupts these functions, resulting in increased intestinal permeability, bacterial translocation, immune activation, and chronic inflammation [120].

Given the critical role of gut microbiota dysbiosis in IBD pathogenesis, dietary interventions targeting microbial modulation—particularly through prebiotics, like KGM—have emerged as a promising therapeutic strategy. As a viscous soluble fiber, KGM effectively strengthens the colonic mucosal barrier [121]. In a study by Chen et al., subjects receiving KGM supplementation (1.5 g/d or 4.5 g/d) alongside a standard low-fiber diet demonstrated significant changes after 21 days. The intervention notably increased fecal levels of *Lactobacillus* and *Bifidobacterium*, enhanced colonic fermentation, and attenuated the reduction in fecal pH. Importantly, KGM supplementation substantially elevated fecal SCFAs concentrations, thereby improving gut ecological health and potentially reducing the risk of IBD [122]. Building on these findings, recent studies have focused on the prebiotic effect of KGM to improve gut microbiota imbalance in IBD patients. Animal models provide mechanistic insights: In a study of dextran sulfate sodium (DSS)-induced murine colitis, Xiao et al. found that KGM, when combined with inulin, promoted the proliferation of beneficial bacteria, such as *Bifidobacterium* and *Lactobacillus*, while suppressing *Clostridium* populations. This intervention simultaneously elevated SCFAs levels and mitigated colitis progression [123]. The therapeutic relevance of these microbial shifts becomes clear when examining their immunological consequences: Notably, *Bifidobacterium* alleviates colitis severity by reducing colonic expression of the pro-inflammatory cytokine IL-1β, thereby attenuating intestinal inflammation [124]. Conversely, certain *Clostridium* species (e.g., *Clostridium difficile*) proliferate during microbiota imbalance, releasing toxins that trigger pseudomembranous colitis and severe diarrhea [125]. Additionally, bacterial metabolism of KGM generates substantial butyrate, a key metabolite with demonstrated therapeutic effects in IBD. At the cellular level, in vitro studies indicate that butyrate promotes normal colonocyte proliferation, enhances tight junction protein transcription (e.g., ZO-1, occludin), and facilitates their redistribution in cell membranes—mechanisms critical for intestinal barrier restoration [126].

Collectively, these findings demonstrate that KGM improves the intestinal microenvironment in IBD patients by modulating gut microbiota composition and elevating SCFA production, ultimately alleviating disease symptoms and pathological damage.

#### 4.2.2. Colorectal Cancer

CRC ranks among the most prevalent malignant tumors globally, with its development involving a multifactorial, multistage process [127]. The gut microbiota, as the most complex microecosystem in humans, plays crucial roles not only in digestion, metabolism, and immune regulation but also in CRC pathogenesis [128]. Growing evidence indicates that bacterial-derived toxic metabolites can induce DNA damage, disrupt cell cycle progression, trigger aberrant immune responses, and compromise intestinal barrier integrity—collectively fostering a tumorigenic microenvironment [129,130]. Through a comprehensive meta-analysis of 526 metagenomic samples, Dai et al. identified 7 bacteria species consistently enriched in CRC patients, namely *Bacteroides fragilis*, *Fusobacterium nucleatum*, *Porphyromonas asaccharolytica*, *Parvimonas micra*, *Prevotella intermedia*, *Alistipes finegoldii*, and *Thermanaerovibrio acidaminovorans* [131]. Complementary research by Xue et al. has shown that specific microbiota members (including *Escherichia coli*, *Enterococcus*, *Bacteroides*, and *Clostridium*), exacerbate CRC progression by promoting 1,2-dimethylhydrazine-induced abnormal crypt foci formation [132]. Given the established link between dysbiotic gut microbiota and CRC pathogenesis, interventions capable of modulating microbial composition and metabolic activity—such as prebiotics—hold promise for mitigating oncogenic processes. Among these, KGM emerges as a potent candidate due to its dual capacity to reshape microbial communities and suppress carcinogenic metabolites.

As a natural prebiotic, KGM can regulate the structure of gut microbiota and produce beneficial metabolites after ingestion, which plays an important role in alleviating CRC. To demonstrate this clinically, Wu et al. found that KGM capsules (4.5 g/d) could significantly reduce the production of precancerous markers of CRC after four weeks of supplementation in subjects with a low-fiber diet. Notably, only the levels of fecal *Bifidobacterium faecalis* and *Lactobacilli* of subjects supplemented with KGM capsules increased significantly, and the total amount of intestinal bacteria increased substantially [133]. These microbial changes highlight KGM’s dual role: it not only improves the imbalance of gut microbiota and increases the number of beneficial bacteria but also triggers downstream metabolic effects. Moreover, KGM supplementation significantly reduced the concentration of secondary bile acids in the fecal water phase of the subjects. SCFAs are produced by the fermentation and metabolism of KGM by gut microbiota. The acidic fecal environment caused by SCFAs contributes to the precipitation of hydrophilic deconjugated bile acids and inhibits the enzymatic conversion of primary bile acids to secondary bile acids [134]. The clinical significance of this bile acid modulation becomes clear when considering their carcinogenic potential: Secondary bile acids (deoxycholic acid, DCA; lithocholic acid, LCA) activate carcinogenic signaling pathway, inhibit the activity of farnesoid X receptor (FXR), downregulate the expression of its downstream target genes (e.g., FGF19), disrupt bile acid homeostasis, and activate the Takeda G protein-coupled receptor 5 (TGR5) receptor to trigger the cAMP-PKA-CREB pathway, promoting the release of pro-inflammatory factors (e.g., COX-2, IL-6) and accelerating tumor cell proliferation (it should be noted that TGR5 seems to have different effects in different cell and tissue backgrounds). Beyond inflammation, through the Wnt/β-catenin pathway, they also drive oncogene (e.g., c-Myc) transcription, inducing CRC [135,136]. Furthermore, secondary bile acids induce the production of reactive oxygen species (ROSs), leading to oxidative damage of tight junction proteins and increasing the risk of CRC [137]. These findings further suggest that KGM has a protective effect against the occurrence of human CRC.

In addition, many studies have found that KGM supplementation can also effectively inhibits the proliferation of potential pathogenic bacteria, such as *Fusobacterium nucleatum* and some *Escherichia coli* strains, reducing their damage to the intestinal barrier and the accumulation of carcinogenic metabolites [138,139,140]. This suppression of harmful bacteria is particularly significant in CRC prevention, as *Fusobacterium nucleatum* is a pathogenic bacterium closely related to CRC, which can contribute to CRC development by activating inflammatory signaling pathways (e.g., NF-κB) and promoting tumor cell proliferation [141]. Beyond direct pathogen inhibition, KGM indirectly inhibits the development of CRC by restoring gut microbiota diversity and improving the intestinal microenvironment. This is critical because CRC patients exhibit significantly reduced gut microbiota diversity [142], whereas KGM intake increases both α-diversity and β-diversity of the flora, promoting a healthier microbial composition [143].

**Figure 5:**
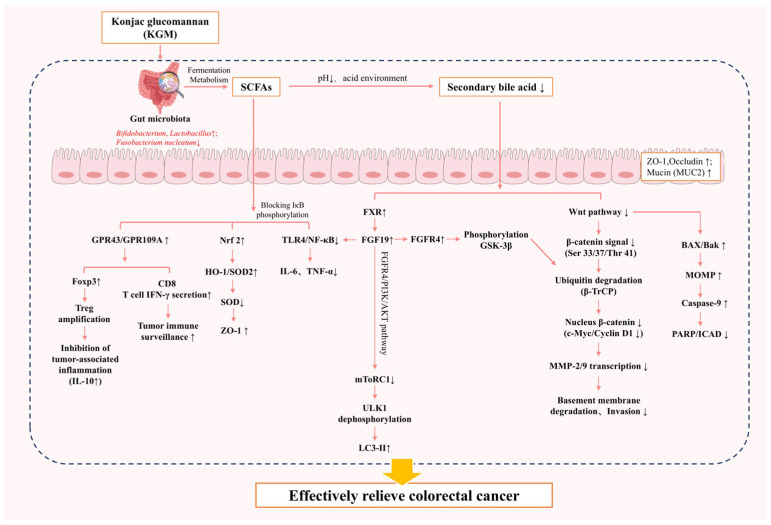
Mechanism of KGM in alleviating colorectal cancer; “↑” indicates increases, while “↓” indicates decreases; arrows denote activation steps.

Collectively, these findings demonstrate that KGM exerts its protective effects against CRC through the following multifaceted approach: modulating gut microbiota composition (enriching beneficial taxa while suppressing pathogens), reducing carcinogenic metabolites (e.g., secondary bile acids and ROSs), and restoring microbial diversity—thereby disrupting key oncogenic pathways and reinforcing intestinal barrier integrity (Figure 5).

### 4.3. Immunoregulation

The immune system is an important defense mechanism that resists pathogen invasion and maintains internal environment stability [144]. Immune system balance is crucial, as excessive activation may cause autoimmune diseases, while dysfunction increases infection and cancer risks [145]. Recent studies have reported that the dynamic interactions between gut microbiota and the host immune system are essential for maintaining intestinal homeostasis and suppressing inflammation [146]. Gut microbiota imbalance or detrimental changes in microbial composition can disrupt immune responses, leading to inflammation and oxidative stress [147]. In DSS-induced colitis mice, *Escherichia coli* abundance significantly increased, promoting systemic pathogen circulation and inflammasome activation [148]. Additionally, numerous studies have found that increased *Clostridium difficile* abundance can cause severe intestinal inflammation and exacerbate inflammatory factor-induced damage [149,150].

Against this backdrop, KGM emerges as a potent immunomodulatory prebiotic, capable of reshaping gut microbiota composition to suppress pro-inflammatory responses while reinforcing protective immune mechanisms. Liu et al. demonstrated that supplementation significantly increased the diversity of beneficial gut microbiota, including *Lactobacillus*, *Lachnoclostridium*, *Alloprevotella*, and *Blautia*, thereby improving microbial diversity. This microbial restructuring directly translates to functional benefits, promoting intestinal homeostasis by reducing systemic absorption of bacterial toxins (e.g., LPS) and mitigating chronic inflammation [29]. Notably, KGM markedly elevated the relative abundance of *Lachnospiraceae* and *Akkermansia muciniphila*, both known for their ability to produce SCFAs. SCFAs serve as pivotal mediators of KGM’s immunomodulatory effects, as they are vital for intestinal energy supply, mucosal barrier integrity, gut motility regulation, and immune function [97]. Mechanistically, SCFAs exert anti-inflammatory effects by activating macrophages and dendritic cells, thereby suppressing pro-inflammatory cytokines (e.g., TNF-α, IL-12, and IL-6) [151]. Beyond SCFA-dependent pathways, propionate—generated through KGM’s microbial fermentation—activates macrophages via GPR43 receptor signaling, enhancing antimicrobial peptide secretion and resolving inflammatory states [152]. Equally important are KGM’s broader impacts on microbial ecology, as recent studies have revealed that KGM-fed HFD mice exhibited increased *Parabacteroides distasonis* abundance, with its metabolites participating in bile acid metabolism and immune cell differentiation [153]. Critically, KGM’s benefits extend to pathogen suppression, e.g., inhibiting *Odoribacter* sp. Z80, which reduces inflammatory factor release and further supports immune balance [78].

Based on the above multiple mechanisms, KGM effectively modulates gut microbiota structure and demonstrates significant immunoregulatory properties, offering novel strategies for managing immune-related disorders.

### 4.4. Nervous System Diseases

Nervous system diseases refer to neurological or mental disorders resulting from structural or functional abnormalities in the brain or spinal cord due to various factors [154]. Accumulating evidence indicates a strong association between gut microbiota dysbiosis and various neurological diseases [155,156].The gut–brain axis, a bidirectional communication network linking the central nervous system and the gut microbiome, is critically involved in neurological diseases, such as schizophrenia, depression, autism spectrum disorders, AD, and PD [157,158]. Notably, significant alterations in gut microbiota composition and relative abundance have been observed in these disorders [159]. For instance, Marcus M. Unger et al. reported that PD patients exhibit a marked increase in Enterobacteriaceae, reduced *Prevotella* levels, and significantly decreased fecal SCFAs concentrations compared to healthy individuals [160]. Similarly, studies on AD-associated dementia revealed a decline in Firmicutes and *Bifidobacterium*, alongside an increase in Bacteroidetes [161]. Interestingly, a similar reduction in Firmicutes has been documented in T2DM patients [162], and both diabetes and insulin resistance are recognized risk factors for AD [163]. This correlation may stem from insulin resistance’s association with impaired brain glucose metabolism and accelerated amyloid deposition in asymptomatic middle-aged individuals [164].

KGM has high biocompatibility and significant health benefits, with emerging evidence supporting its therapeutic potential in neurological disorders. Specifically, KGM is widely considered a prebiotic that can be fermented by intestinal anaerobic bacteria [165]. Its metabolites (e.g., SCFAs) serve as signaling molecules in the “gut-brain” axis to improve the pathogenesis of mental illness through epigenetic regulation, neuroinflammatory regulation, blood–brain barrier maintenance, and brain metabolic regulation [166,167]. Gou et al. established an AD mice model and administered KGM at a dose of 800 mg/kg. After 12 weeks of KGM supplementation, the abundance of *Prevotella* sp. CAG:485, Muribaculaceae bacterium Isolate-114 (HZI), and *Parabacteroides distasonis* in the intestinal tract of mice was significantly increased. The treatment significantly inhibited the growth of *Enterorhabdus caecimuris*, *Odoribacter* sp. Z80, and bacterium 1XD8-76, thereby improving the gut microbiota structure in AD mice and effectively alleviating AD progression [78]. SCFAs play a pivotal role in KGM’s neuroprotective effects. Following fermentation and catabolism of intestinal anaerobic bacteria (*Bifidobacterium*, *Lactobacillus*), KGM significantly increased the content of acetate, butyrate, and isobutyrate while decreasing isovalerate levels. These metabolites exhibit strong associations with key AD biomarkers. Acetate showed positive correlations with brain-derived neurotrophic factor (BDNF) and the novel object recognition response index (NORF-RI), and negative correlations with Aβ1-40, Aβ1-42, p-Tau181, and p-Tau217. The mechanistic link between SCFAs and neuroprotection is well established. BDNF inhibits GSK3β activity by activating TrkB receptors, initiating the PI3K/Akt and MAPK/ERK signaling pathways, and thereby reducing Aβ toxicity, decreasing Tau protein hyperphosphorylation, enhancing neuronal survival and synaptic plasticity, and ultimately improving memory impairment and nerve damage in AD [168,169]. KGM’s reduction of harmful metabolites provides additional therapeutic benefits. Studies report that high concentrations of isovalerate can lead to motor and cognitive dysfunction [170]. By lowering isovalerate production, KGM indirectly mitigates cognitive decline. Similar neuroprotective effects are observed in PD. Butyrate improves motor dysfunction by inhibiting neuroinflammation and protecting dopaminergic neurons [171]. KGM’s promotion of bacteria further enhances its neurological benefits. Numerous studies confirm that KGM intake can significantly increase *Bifidobacterium*, *Lactobacillus*, and *Roseburia* abundance [35,143]. These bacteria directly influence neurotransmitter balance. *Bifidobacterium* can synthesize serotonin 5-hydroxytryptamine (5-HT) and γ-aminobutyric acid (GABA) [172], alleviating depression and anxiety in AD patients though 5-HT elevation [173]. *Lactobacillus* can produce GABA [174], reducing neuronal excitability and seizure frequency in epilepsy [175]. Additionally, *Lactobacillus* indirectly improves neurological diseases by regulating intestinal barrier function, reducing endotoxin and harmful metabolite penetration, and decreasing systemic and central nervous system inflammation [6].

Therefore, based on the above-mentioned mechanism of KGM in improving neurological diseases, it can be used as a key dietary intervention to enhance neurological health by modulating gut microbiota, offering novel strategies for the prevention and treatment of neurological disorders.

## 5. Limitations of KGM

Although KGM demonstrates significant effects in regulating blood glucose and lipid levels, its practical application faces several limitations related to digestive tolerance, potential adverse effects, nutrient interference, and individual differences. As a high-viscosity water-soluble dietary fiber, excessive KGM intake may cause gastrointestinal disturbances. Upon hydration, KGM expands to tens or even hundreds of times its original volume, forming thick, gel-like masses in the intestinal tract. This property poses particular risks for individuals with impaired intestinal motility (e.g., postoperative patients and elderly populations), potentially leading to mechanical obstruction and significantly increasing the likelihood of abdominal distension or intestinal blockage [11,15]. Additionally, incomplete fermentation of KGM and other indigestible polysaccharides (e.g., arabinose) by gut microbiota can elevate lumen osmotic pressure, triggering excessive fluid secretion [176]. Clinical trials have reported adverse effects in some individuals: Among 195 obese or diabetic patients receiving 7.8 g/day of KGM (equivalent to about 110 mg/kg/day) for 16 weeks, 13.4% experienced flatulence and abdominal discomfort [177]. Similarly, a meta-analysis by Onakpoya et al. on KGM as a weight-loss supplement found that daily doses between 1.2–10 g (17–143 mg/kg/day) for 3–12 weeks led to various symptoms, such as bloating, loose stools, diarrhea, and abdominal pain, in some participants [178].

At the nutrient absorption level, the strong gel-forming properties of KGM may impair nutrient bioavailability. Its anionic polysaccharide structure exhibits chelating effects on essential minerals, such as calcium, iron, and zinc, potentially reducing their absorption [179]. Additionally, the intestinal gel network formed by KGM can trap fat-soluble vitamins (e.g., vitamins A, D, and E), diminishing their absorption efficiency in the small intestine [180]. This effect may be particularly significant in elderly individuals or those with reduced digestive enzyme secretion, potentially exacerbating micronutrient deficiency risks. Notably, KGM’s interactions with pharmaceuticals require clinical consideration [181,182]. Shima et al. demonstrated that plasma glibenclamide concentrations were significantly lower at 30, 60, 90, and 150 min after co-administration of 3.9 g konjac flour (containing KGM) and 2.5 mg glibenclamide, compared to a control group. This suggests that KGM slows down and reduces the absorption of hypoglycemic drugs [183]. Therefore, a temporal separation of at least 2 h between KGM intake and medication administration is recommended to minimize interaction risks. Finally, KGM’s physiological effects exhibit considerable interindividual variability. Due to the highly personalized composition of gut microbiota, some individuals may show limited fermentative responses to KGM, resulting in diminished prebiotic benefits.

In conclusion, while KGM represents a functionally valuable dietary fiber, its consumption requires careful regulation and individualized dosage adjustments based on personal health status to minimize potential adverse effects.

## 6. Conclusions

As a natural functional polysaccharide, KGM exhibits significant potential in metabolic regulation, intestinal health maintenance, immune modulation, and neuroprotection, attributed to its unique physicochemical properties and broad-spectrum bioactivities. The core mechanism underlying these benefits lies in gut microbiota-mediated fermentation and biotransformation. Through selective promotion of beneficial bacterial proliferation while suppressing potential pathogens, KGM optimizes microbial ecological balance and stimulates production of key metabolites (e.g., SCFAs). These processes collectively exert multifaceted effects, including anti-inflammatory activity, restoration of the epithelial barrier, regulation of glucose/lipid metabolism, and neurological functions.

Nevertheless, the practical application of KGM is currently hindered by several limitations. Interindividual variations in gut microbiota composition may lead to inconsistent efficacy; potential gastrointestinal intolerance (e.g., bloating or diarrhea) could restrict its dosage range; and risks of nutritional interference (e.g., interactions with other dietary components) require further clarification. These challenges highlight the need for targeted advancements in subsequent research. Priority areas for future studies should focus on the following three aspects: (1) Precise dosage optimization, integrating individual microbiota profiles and physiological characteristics to establish personalized administration strategies, thereby addressing interindividual efficacy variations; (2) development of strain-specific microbiota modulation approaches, such as combining KGM with probiotic strains that synergistically enhance its prebiotic effects, to strengthen its selective regulatory capacity on beneficial bacteria; (3) exploration of synergistic interactions with complementary dietary components (e.g., polyphenols or dietary fibers), aiming to mitigate gastrointestinal intolerance and improve nutritional compatibility.

Collectively, KGM is a natural bioactive compound with prominent prebiotic properties and pleiotropic health benefits. With targeted solutions to current limitations, it holds substantial promise for translation into preventive medicine and innovation in functional foods, contributing to personalized nutrition and public health promotion.

## Figures and Tables

**Table 1:** Effects of KGM on gut microbiota and health.

Disease Type	Dosage	Experimental Subject	Microbiota Influence	Others’ Influence	Conclusions	Reference
Obesity	400 mg/kg	C57Bl/6J	*unclassified_f_Lachnospiraceae*↑, *norank_f_Lachnospiraceae*↑, *Blautia*↑, *Romboutsia*↑, *Colidextribacter*↑, *norank_f_Oscillospiraceae*↑, *Lachnospiraceae_NK4A136*↑, *Faecalibacterium*↓, *Muribaculaceae*↓	Body weight↓, fat mass↓, FBG↓, insulin resistance↓; appetite regulator GLP-1 and intestinal hormone PYY↑; blood lipid levels TG, TC and LDL-C↓, HDL-C↑; inflammatory factors TNF-α, IL-1β, and IL-6↓; expression of lipid metabolism genes LDLR, GCK, and G-6-pase mRNA↑; SCFAs↑	Konjac dietary fiber (KGM) intake reduces body weight in obese mice, improves glucose and lipid metabolism homeostasis, and modulates gut microbiota composition.	[71]
Obesity	100 g/kg	C57Bl/6J	*Bifidobacterium*↑, *Lactobacillus*↑, *Alistipes*↑, *Clostridium*_XlVa↑, *Blautia*↓, *Allobaculum*↓, *Saccharibacteria*↓, *Enterorhabdus*↓, *Coprococcus*↓	Inguinal fat↓, brown fat↑; food intake and energy intake↓, energy consumption and heat production↑; leptin and adiponectin↓, GLP-1↑; inflammatory factors TNF-α, IL-6, and IL-1β↓; repair of intestinal epithelial barrier injury, Claudin-1 and Occludin protein expression↑; expression of *Agrp*, *Npy*, and *Orx* in hypothalamus of obese mice↓, *Cart* expression↑; SCFAs↑	KGM modulates energy balance via the gut microbiota-brain axis, suppressing appetite and alleviating obesity.	[72]
Diabetes	Feed contains 5% KGM	Sprague Dawley male rats	*Muribaculaceae*↑, *Ruminococcus*↑, *Lachnoclostridium*↑, *Romboutsia*↓	FBG, HOMA-IR↓; inflammatory factors TNF-α and IL-6↓; fecal SCFAs↑; expression of *GPR41*, *GPR43*, and *GPR109A* mRNA↑	KGM effectively lowers blood glucose by modulating gut microbiota composition, increasing SCFA levels, and activating G protein-coupled receptors (GPRCs), thereby alleviating diabetes symptoms.	[73]
Diabetes	Liquid food contains 0.38% KGM	C57Bl/6J	*Bifidobacterium*↑, *Allobaculum*↑, *S24-7*↓, *Helicobacter pylori*↓	Body weight, epididymal fat, and subcutaneous fat↓; FBG, serum insulin level, HOMA-IR index, and postprandial blood glucose↓; blood lipid levels TC, TG, and LDL-C↓, HDL-C↑; serum ALT, AST, ALP, and LDH levels↓, effectively reduced liver injury; hepatocellular lesions↓, liver lipid droplets	The KGM-PCP combination improved body weight, lipid homeostasis, and liver health in T2DM mice by lowering postprandial blood glucose and modulating gut microbiota composition and abundance in the intestinal environment.	[74]
Hyperlipidemia	60 g/kg	Male Golden Syrian hamsters	*Lachnospiraceae*_UCG-006↑, *Parasutterella*↑, *Lachnospiraceae*_NK4A136↓, *Lachnoclostridium*↓, *unclassified_f_Oscillospiraceae*↓, *Adlercreutzia*↓, *Eubacterium_brachy_group*↓, *Gordonibacter*↓	Body weight, liver lipid vacuoles, epididymal fat weight and adipocyte size↓; blood lipid levels TC, TG, and LDL-C↓; SCFAs↑; significantly changed bile acid composition, DCA/LCA↓, GCA/UDCA↑; liver CYP7A1 mRNA expression↑; FXR and sterol 12α-hydroxylase (CYP8B1) mRNA expression↓	KGM primarily modulates gut microbiota activity, which in turn alters bile acid metabolism, ultimately improving blood lipid profiles.	[35]
Hyperlipidemia	100 g/kg	C57Bl/6J	*Akkermansia muciniphila*↑, *Alistipes*↑, *Olsenella*↑, *Bifidobacterium*↑, *Sporobacter*↓, *Allobaculum*↓, *Acetatifactor*↓, *Helicobacter pylori*↓	Lipid levels TC, TG, FAA, and LDL-C↓, HDL-C↑, insulin and leptin levels↓; liver function markers AST and ALT↓, SOD↑; lipid accumulation in IECs↓; fatty acid decomposition protein and gene (FABP1, PPARα, SREBP1 mRNA, and CD36 protein expression)↓; depth of crypt and the number of goblet cells↑; improved mucosal epithelial damage and disorderly loose arrangement of epithelial cells	KGM significantly reduced systemic and intestinal lipid accumulation in obese mice by altering microbial populations linked to lipid absorption and enhancing SCFA production, thereby inhibiting lipid absorption and output in HFD mice.	[32]
IBD	70 g/kg	C57Bl/6J	*norank_f__Muribaculaceae*↑, *Akkermansia muciniphila*↑, *Parabacteroides*↑, *Monoglobus*↑, *Bacteroides*↓, *Blautia*↓, *Escherichia-Shigella*↓, *Colidextribacter*↓, *Salmonella*↓	Serum MDA and H_2_O_2_↓; relative mRNA levels of Nqo1 and Nrf2↑; alleviated colonic oxidative stress; tight junction OCLN and ZO-2 protein expression↑; TNF-α, Ccl8, and Il-10 mRNA↑; INF-β mRNA↓; inflammatory factors Ccl2, Ccl3, Ccl8, and interleukin-1β (Il-1β) mRNA expression↓; reduced inflammatory response; Toll-like receptor (Tlr2, Tlr9) mRNA↓; ratio of phosphorylated (p) -NF-κB/NF-κB↓	KGM alleviates colitis by modulating the gut microbiota and inhibiting the TLR2/NF-κB signaling pathway.	[75]
IBD	25 g/kg	C57Bl/6J	*Lactobacillus*↑, *Bifidobacterium*↑, *Clostridium*↓	Improved the distal colon cross-sectional tissue dysplasia; inflammatory factors TNF-α, IL-6↓, and IL-10↑; tight junction protein ZO-1 and occludin gene expression↑; improved the intestinal barrier damage; fecal SCFAs↑	The KGM-inulin oligosaccharide combination prevents colitis by modulating gut microbiota, strengthening the intestinal barrier, and boosting SCFA production to suppress inflammation.	[76]
CRC	62.5 g/kg	Sprague Dawley male rats	*Bifidobacterium*↑, *Lactobacillus*↑, *Clostridium*↓	Energy intake and body weight↓; fecal β-glucuronidase and mucin activity↓; lithocholic acid ↓, fecal excretion↑; cecal i-butyrate↑	The KGM-inulin combination enriched beneficial gut bacteria, altered microbiota composition, elevated SCFA levels, and improved the intestinal environment, thereby reducing fecal secondary bile acids and lowering CRC risk.	[77]
Immunoregulation	0.4 g/kg	C57Bl/6J	*Lactobacillus*↑, *Lachnoclostridium*↑, *Alloprevotella*↑, *Blautia*↑, *f_Lachnospiraceae*↑, *Akkermansia muciniphila*↑	Pulse oxygen saturation (SpO2) ↑; peripheral blood components RBC, WBC, PLT, HGB, and Lym↑; white pulp area of the spleen, femur, and spleen cells↑, improved ability of bone marrow to produce new blood cells damage; crypt and villus structure of mice were protected; DAO↓; improved intestinal permeability, relieved inflammation; SCFAs (acetate, propionate, and butyrate)↑	KGM boosts gut microbiota abundance, probiotics, and SCFA production, maintains intestinal homeostasis, protects epithelial cells from apoptosis, and reduces inflammation while enhancing immune regulation.	[29]
AD	800 mg/kg	C57Bl/6J	*Prevotella* sp. CAG:485↑, *Muribaculaceae bacterium* Isolate-114 (HZI) ↑, *Parabacteroides distasonis*↑, *Duncaniella freteri*↑, *Alistipes* sp. 56 11↑, *Alistipes* sp. Z76↑, *Muribaculum* sp. NM65 B17↑, *Alistipes* sp. HGB5↓, *Alistipes* sp. CAG:268↓, Bacterium 1XD8-76↓, *Alistipes finegoldii*↓, *Alistipes onderdonkii*↓, *Muribaculaceae bacterium* Isolate-104 (HZI) ↓, *Odoribacter* sp. Z80↓, *Enterorhabdus caecimuris*↓	Identification index (RI)↑; central area crossing ability↑; average speed↓; effectively relieved anxiety; positive area of Aβ1-40 and Aβ1-42↓ effectively inhibited the accumulation of Aβ in hippocampus, and the hyperphosphorylation of Tau protein was inhibited; expression of bdnf↑; brain-derived neurotrophic factor (BDNF)↑; eelative expression of trkb, pi3k, and akt↑; relative expression of gsk3β↓; activated the BDNF/TrkB signaling pathway	KGM may ameliorate AD by modulating gut microbiota composition, elevating SCFA levels, activating the BDNF/PI3K/GSK3β pathway to enhance hippocampal neurogenesis, and reducing Aβ/Tau accumulation.	[78]

Note: Arrows indicate changes in content or proportion, “↑” indicates increases, while “↓” indicates decreases.

## Data Availability

Not applicable.
